# Synthesis and Performance Evaluation of Alginate-Coated Temperature-Sensitive Polymer Gel Microspheres

**DOI:** 10.3390/gels9060480

**Published:** 2023-06-12

**Authors:** Zhaozheng Song, Junhang Hu, Ping Liu, Yili Sun

**Affiliations:** 1 State Key Laboratory of Shale Oil and Gas Enrichment Mechanisms and Effective Development, Beijing 102206, China; hujunhang00@126.com (J.H.);; 2 Research and Development Center for the Sustainable Development of Continental Sandstone Mature Oilfield by National Energy Administration, Beijing 102206, China; 3 College of Science, China University of Petroleum (Beijing), Beijing 102249, China; 4 Oil and Gas Development Management Department, Sinopec Henan Oilfield Branch, Nanyang 473132, China

**Keywords:** polymer microspheres, temperature sensitive material, nano-TiO_2_, plugging performance

## Abstract

With the long-term water-flooding development of the reservoir, the non-homogeneity of the formation is increasing and the reservoir environment is deteriorating; the microspheres used for deep plugging have shown disadvantages, such as poor temperature and salt resistance and faster expansion. In this study, a polymeric microsphere was synthesized that is resistant to high temperature and high salt and can achieve slow expansion and slow release for deep migration. P(AA-AM-SA)@TiO_2_ polymer gel/inorganic nanoparticle microspheres were prepared by reversed-phase microemulsion polymerization using acrylamide (AM) and acrylic acid (AA) as monomers, 3-methacryloxypropyltrimethoxysilane (KH-570)-modified TiO_2_ as the inorganic core, and sodium alginate (SA) as a temperature-sensitive coating material. Through single-factor analysis of the polymerization process, the optimal synthesis conditions were determined as follows: the oil(Cyclohexane)-water volume ratio was 8:5, the emulsifier mass ratio (Span-80:Tween-80) was 3:1 (10 wt% of the total system amount), the stirring speed was 400 r/min, the reaction temperature was 60 °C, and the initiator (ammonium persulfate and sodium bisulfite) dosage was 0.6 wt%. The size of the dried polymer gel/inorganic nanoparticle microspheres prepared by the optimized synthesis conditions was 10~40 μm with uniform particle size. The observation of P(AA-AM-SA)@TiO_2_ microspheres reveals that the Ca elements are uniformly distributed on the microspheres, and FT-IR indicates that the synthesized product is the target product. TGA shows that the polymer gel/inorganic nanoparticle microspheres have better thermal stability after the addition of TiO_2_, with a larger mass loss at 390 °C, which can adapt to the medium-high permeability reservoir environment. The thermal and aqueous salinity resistance of the P(AA-AM-SA)@TiO_2_ microspheres was tested, and the cracking temperature of P(AA-AM-SA)@TiO_2_ microsphere temperature-sensitive material was 90 °C. It still has favorable water absorption and swelling performance under the sodium salt concentration of 2.5 × 10^4^ mg/L and can tolerate calcium salt up to 2.0 × 10^4^ mg/L. Plugging Performance Test results show that the microspheres have good injectability between the permeability of 1.23 and 2.35 μm^2^ and good plugging effect near the permeability of 2.20 μm^2^. At high temperature and high salinity, P(AA-AM-SA)@TiO_2_ microspheres have a remarkable effect on profile control and water shutoff, the plugging rate reaches 95.3%, and the oil recovery rate is increased by 12.89% compared with water flooding, achieving the effect of slow swelling and slow release.

## 1. Introduction

With the rapid growth of the global economy, the increasing extraction of crude oil and decreasing extractable energy have posed a great challenge to the world [1]. Most domestic and foreign oil fields are developed to a high water-bearing stage, and long-term water erosion exacerbates the inhomogeneity of the reservoir, leading to water flushing and even water monolayer breakthrough [2]. The injected water will flow into the producing wells along the dominant channel of water flow, leading to the problems of premature water, small ripple effect, and low oil production during the development of the oil field. Oil in low-permeability zones cannot be recovered in the formation. As a result, the efficiency of water flooding is greatly reduced and the effectiveness of various types of repellents cannot be fully utilized [3,4,5]. In this case, most of the remaining oil (about 65% to 77%) remains confined in the subsurface [6]. Therefore, closing the high-permeability zone and increasing the water flooding wave area is the key to improving the recovery of nonhomogeneous oil reservoirs.

To achieve this goal, various materials have been applied to profile control and enhance oil recovery, such as cross-linked polymer gels, weak gels, dispersion gels (DPG), preformed particle gels (PPG), and polymer microspheres. [7,8,9,10,11]. Among them, polymer microspheres are a kind of prefabricated microgel particle. Polymer microspheres are rapidly developing in the field of profile modification, with a wide range of applications, and are receiving a lot of attention [12,13,14,15]. These elastic spheres with micron and nano dimensions can block highly permeable channels by adsorption, aggregation and bridging, and divert the subsequent water flow to untouched areas. At the same time, they can cross the pore throat through the inherent elastic deformation ability and continuously migrate to the deep part of the reservoir to increase the permeability resistance of the high water-bearing zone [14]. Bai et al. [16] successfully prepared a polymer microsphere using acrylamide (AM) and 2-acrylamide-2-methylpropanesulfonic acid (AMPS) as copolymer monomers. The results showed that these aqueous dispersions of microspheres had high viscosity and good dispersion stability, with a median particle size of 1 μm.

However, the general dissection agent only functions as a sealer in the near-well zone and has little transport capacity [17]. Conventional polymer microspheres also have some inherent defects due to their sensitivity to harsh reservoir conditions (e.g., high temperature, high salinity, and high mechanical shear) and lose their ability to regulate profiles [4,14]. Therefore, improving the high temperature and high salt resistance of these microgel particles, as well as the slow expansion and slow-release performance, is an essential requirement for profile control and water shutoff. Yao et al. [18] used N,N-methylenebisacrylamide (MBA) as a cross-linking agent and carried out polymerization reaction to prepare a plugging agent, which has certain resistance to high temperature and high salt, as well as water absorption and swelling properties. Yang [19] used reversed-phase suspension polymerization to prepare a polymer microsphere for oil flooding in oilfields using AM, acrylic acid (AA), and AMPS as monomers, MBA as cross-linker, ammonium persulfate as initiator, and Span-80 and Tween-60 as dispersants, and its temperature and salt resistance were improved compared with conventional microspheres.

Due to the increasing demand for the performance of profile control agent, it is difficult to adapt a single material to the actual needs, and inorganic/organic composites are gradually coming into view [20,21,22,23]. Inorganic/organic composites have the stability, dispersibility, temperature and salt resistance, and rigidity of inorganic materials and the elasticity, polymerizability, and ductility of organic materials. The composite microspheres combine the advantages of both and have good application prospects. TiO_2_ particles are probably the most commonly used inorganic nanomaterials and have attracted attention in many fields [24]. Nano-TiO_2_ contains titanium-oxygen bonds and exhibits strong polarity, and its surface can adsorb water, which is further decomposed into hydroxyl groups. Using these hydroxyl groups as reaction sites, surface modification can be carried out on nano-TiO_2_ to introduce nonpolar functional groups, which enhances its dispersibility and compatibility with polymers. 3-methacryloxypropyltrimethoxysilane (KH-570) is a good surface modifier; the hydroxyl group at one side can be connected to the metal oxide and the carbon-carbon double bond at the other side can be involved in the polymerization reaction. The modified TiO_2_ is less likely to agglomerate, has better interfacial compatibility with polymers [25], and has potential for application in the synthesis of polymer gel/inorganic nanoparticle microspheres. The incorporation of inorganic metal particles into the polymer can effectively improve the shear resistance of the polymer microspheres, making it easier for them to migrate to the deeper formation. Huang et al. [26] synthesized the composite material AR/SiO_2_ using inorganic nanomaterial SiO_2_ and organic material acrylic resin, which is in rubbery state in the subsurface at high temperature and also has good shear resistance due to the presence of inorganic particles, which can be squeezed into the shale pores to form a seal under the action of pressure difference. Using nanoparticles in a drilling fluid mud, Al-yasiri et al. [27] developed a xanthan gum-SiO_2_ mixture to enhance water-based mud (WBM) formulation. The modified drilling fluid had a higher yield point compared to the conventional WBM.

Another problem encountered in deeper transfer is high temperature. In higher temperature environments, polymer microspheres tend to absorb water and swell early to form hydrogels, which are difficult to migrate to deeper formations. Sodium alginate (SA) can be prepared as a hydrogel film due to its good biocompatibility, low toxicity, low extraction cost, and easy binding to divalent metal ions, which are widely used in the biomedical field [28,29]. In addition, SA exhibits hydrophobicity at low temperature. Exploiting this property, SA can be used to cover polymers and cross-link them via calcium ions to form a network, which improves the premature water swelling of the polymer. When the temperature is high, SA exhibits hydrophilic properties. SA absorbs water and ruptures, exposing the polymer inside, which further absorbs water and expands to achieve plugging. Bai et al. [30] prepared a granular polyacrylamide-based temporary plugging agent using sodium alginate as an auxiliary reinforcing material, and the results showed better solubility of the temporary plugging agent at higher temperatures.

In this study, a temperature-sensitive polymer microsphere was prepared by reversed-phase microemulsion polymerization using AA, AM as monomer, and nano-TiO_2_ as inorganic composite material, and the polymer microsphere was coated with SA. The optimal synthesis route is determined by regulating the oil-water ratio, emulsifier ratio, stirring speed, temperature, and initiator dosage to optimize the polymer yield and particle size. The structures of the microspheres were analyzed by SEM, EDS, FTIR, and TGA. The effects of temperature and mineralization on the water absorption and swelling properties of polymer microspheres were studied. The membrane cleavage temperature, rheology, and viscoelasticity of the coated microspheres were investigated. Finally, the injection performance of polymer microspheres, their ability to seal the formation, and their ability to enhance recovery were evaluated by plugging performance tests.

## 2. Results and Discussion

### 2.1. Optimization of Synthesis Conditions

#### 2.1.1. Oil-Water Ratio

**Figure 1:**
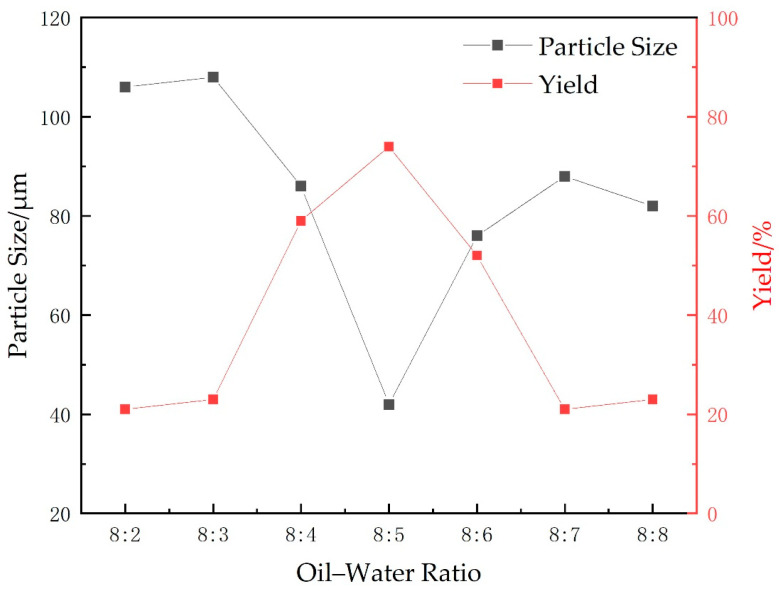
Effects of different oil-water ratios on the yield and particle size of microspheres.

The effects of different oil(Cyclohexane)-water volume ratios on the particle size and yield of microspheres were investigated while maintaining the cross-linking agent at 0.75 g (15 wt% of the total monomers), the emulsifier mass ratio at 3:1 (amount of emulsifier was 10 wt%), the total initiator of ammonium persulfate (APS) and sodium bisulfite (SHS) at 0.6 wt% of the total monomers, the stirring speed at 300 r/min, the temperature at 40 °C, the monomer mass at 5 g (AA:AM mass ratio of 2.8:2.2, 5.6 wt% for AA, 4.4 wt% for AM), and the pH value was adjusted to neutral. The experimental results are shown in Figure 1.

It can be seen from Figure 1 that, when the oil-water ratio is too large or too small, the monomers are prone to implosion and uniformly dispersed microspheres cannot be obtained. This is because the oil-water ratio affects the dispersibility of the two phases. When the proportion is moderate, the oil-water system forms a stable microdispersion system under the emulsifier complex system. Monomers undergo polymerization reaction in these microdispersion systems, forming smaller-diameter polymer microspheres. Therefore, choosing the appropriate oil-water ratio, the polymer is better for initiating polymerization. When the oil-water ratio is 8:5, the particle size of the microspheres is the smallest, the median particle size is about 40 μm, and the highest reaction yield can reach more than 70%.

#### 2.1.2. Emulsifier Ratio

**Figure 2:**
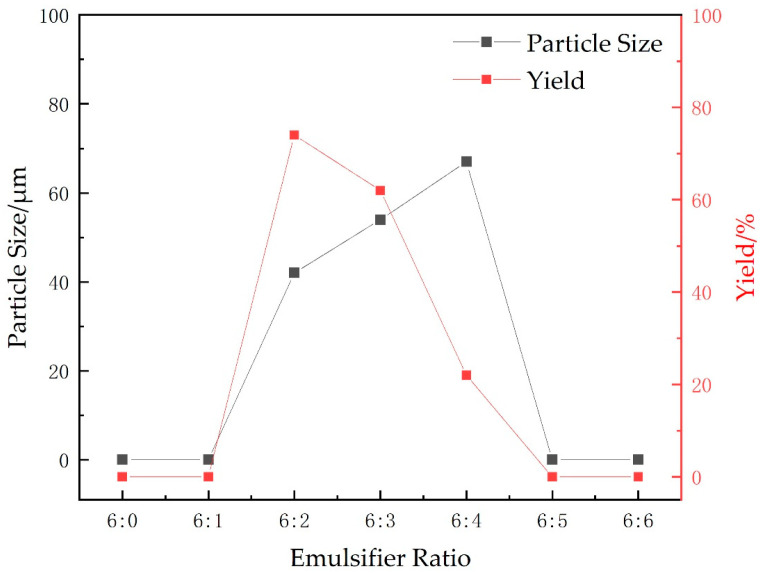
Effects of different emulsifier ratios on yield and particle size of microspheres.

The effects of different emulsifier ratios on the particle size and yield of microspheres were investigated while maintaining the cross-linking agent at 0.75 g (15 wt%), the ratio of oil-water at 8:5, the total initiator (APS + SHS) at 0.6 wt% of the total monomers, the stirring speed at 300 r/min, the temperature at 40 °C, the monomer mass at 5 g (AA:AM mass ratio of 2.8:2.2), and the pH value was adjusted to neutral. The experimental results are shown in Figure 2.

Hydrophilic lipophilic balance (HLB) reflects the combined hydrophilic and lipophilic balance of the emulsifier, and its value is related to the total hydrophilic and lipophilic groups of the system. Emulsifier ratio affects its HLB value, which is crucial for stabilizing the reaction system. By observing the experimental phenomena, an inappropriate emulsifier ratio can lead to an increase in system viscosity during the polymerization process, resulting in turbidity of the product and a yellow-brown solid state after demulsification, making it difficult to obtain the target product. As shown in Figure 2, products can be obtained within the range of emulsifier ratios of 3:1 to 3:2, with suitable microsphere particle size and higher yield at an emulsifier ratio of 3:1. Therefore, the optimal emulsifier ratio is 3:1.

#### 2.1.3. Stirring Rate

**Figure 3:**
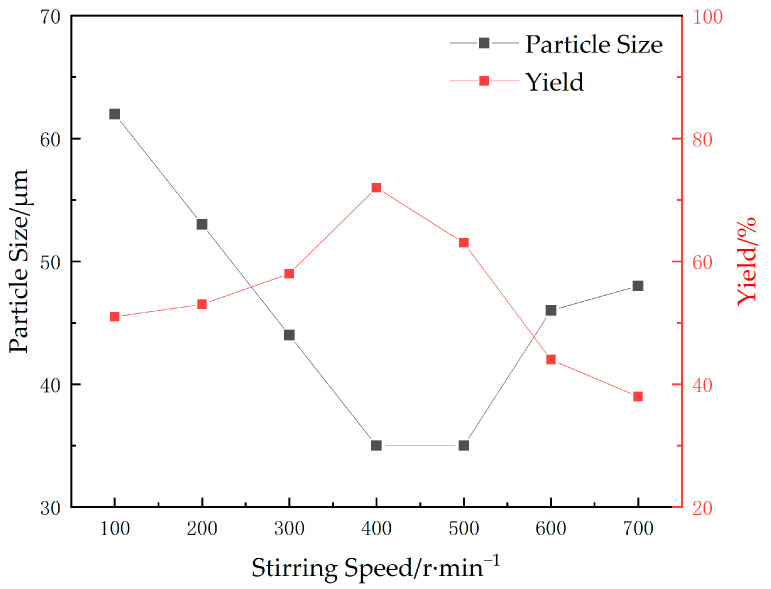
Effects of different stirring speeds on yield and particle size of microspheres.

The effects of different stirring rates on the particle size and yield of microspheres were investigated while maintaining the cross-linking agent at 0.75 g (15 wt%), the oil-water ratio at 8:5, the emulsifier ratio at 3:1 (10 wt%), the total initiator (APS + SHS) at 0.6 wt% of the total monomers, the temperature at 40 °C, the monomer mass at 5 g (AA:AM mass ratio of 2.8:2.2), and the pH value was adjusted to neutral. The experimental results are shown in Figure 3.

As depicted in Figure 3, an investigation into the impact of stirring rate on reversed-phase microemulsion polymerization reveals that microspheres prepared at low agitation rates are inadequate. This is attributed to reduced collision frequency between free radicals and reactive monomers within the microemulsion, resulting in prolonged microsphere formation and larger particle size. Moreover, inadequate stirring causes irregular dispersion of the oil-water phase, thereby impeding complete reaction. A high stirring speed may facilitate homogeneous mixing of the oil-water phases, promoting the occurrence of polymerization reactions. However, excessive agitation can lead to an unstable reaction system, causing aggregation of different polymer chains and enlargement in particle size. Moreover, rapid free-radical release and premature chain termination may also contribute to a low yield of the reaction. When the stirring rate is set at 400 r/min, smaller microspheres can be obtained.

#### 2.1.4. Polymerization Temperature

**Figure 4:**
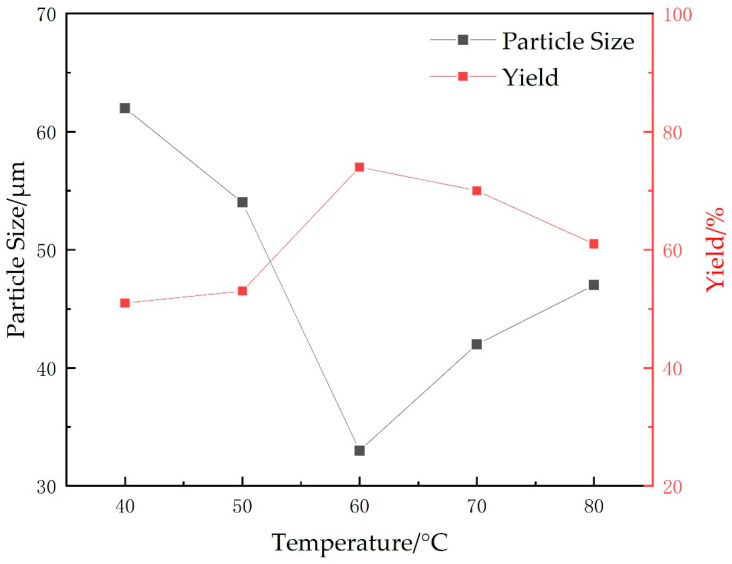
Effects of different temperatures on yield and particle size of microspheres.

The effects of different temperatures on the particle size and yield of microspheres were investigated while maintaining the cross-linking agent at 0.75 g (15 wt%), the oil-water ratio at 8:5, the emulsifier ratio at 3:1 (10 wt%), the total initiator (APS+SHS) at 0.6 wt% of the total monomers, the stirring speed at 400 r/min, the monomer mass at 5 g (AA:AM mass ratio of 2.8:2.2), and the pH value was adjusted to neutral. The experimental results are shown in Figure 4.

From Figure 4, it can be observed that, at low temperatures, the reaction temperature is lower than the decomposition temperature of the initiator, leading to a lower concentration of active free radicals and slower chain growth rate, resulting in reduced productivity. As the temperature rises above the decomposition temperature of the initiator, higher concentration of active free radicals facilitates a more complete reaction, and the exothermicity of the reaction further accelerates the polymerization rate. However, as the temperature continues to rise, the reaction becomes difficult to control and may result in implosion. The productivity of the reaction decreases and the uniformity of the product particles becomes slightly worse, while their size increases slightly. Therefore, the optimal reaction temperature is determined to be 60 °C for achieving the highest yield and optimal particle size of microspheres.

#### 2.1.5. Dosage of Initiator System

**Figure 5:**
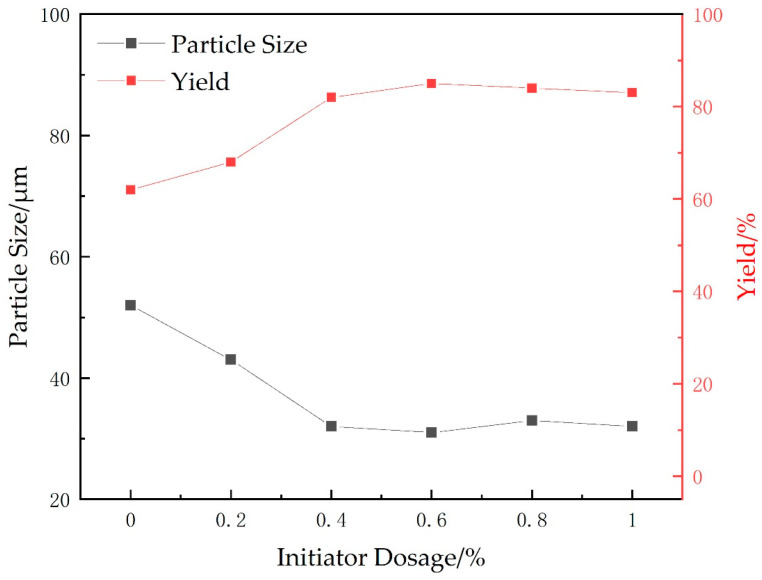
Effects of different initiator dosages on yield and particle size of microspheres.

The effects of different initiator dosages (APS:SHS ratio of 1:1) on the particle size and yield of microspheres were investigated while maintaining the cross-linking agent at 0.75 g (15 wt%), the oil-water ratio at 8:5, the emulsifier ratio at 3:1 (10 wt%), the stirring speed at 400 r/min, the temperature at 60 °C, the monomer mass at 5 g (AA:AM mass ratio of 2.8:2.2), and the pH value was adjusted to neutral. The experimental results are shown in Figure 5.

As can be seen from Figure 5, when the initiator concentration is lower than 0.6 wt%, the microsphere particle size becomes smaller and the yield increases with the increase in initiator. This is mainly due to the low content of initiator; the reaction system can participate in the reaction of fewer free radicals and the chain initiation is slow, resulting in large particle size and low yield. When the initiator content increases, the number of free radicals gradually increases and the yield gradually rises. When the initiator exceeds 0.6 wt%, the chain polymerization reaction is more thorough, and continuing to increase the amount of initiator will not have an effect on the reaction. Therefore, the initiator dose of 0.6 wt% was chosen for the polymerization reaction.

Based on the above single-factor analysis, the optimal conditions for the synthesis of polymer microspheres were as follows: oil-water ratio of 8:5; emulsifier ratio of Span 80:Tween 80 of 3:1 (10 wt%); stirring rate of 400 r/min; reaction temperature of 60 °C; and total initiator (APS:SHS ratio of 1:1) of 0.6 wt%.

### 2.2. Material Characterization

#### 2.2.1. Infrared Spectrum Analysis

**Figure 6:**
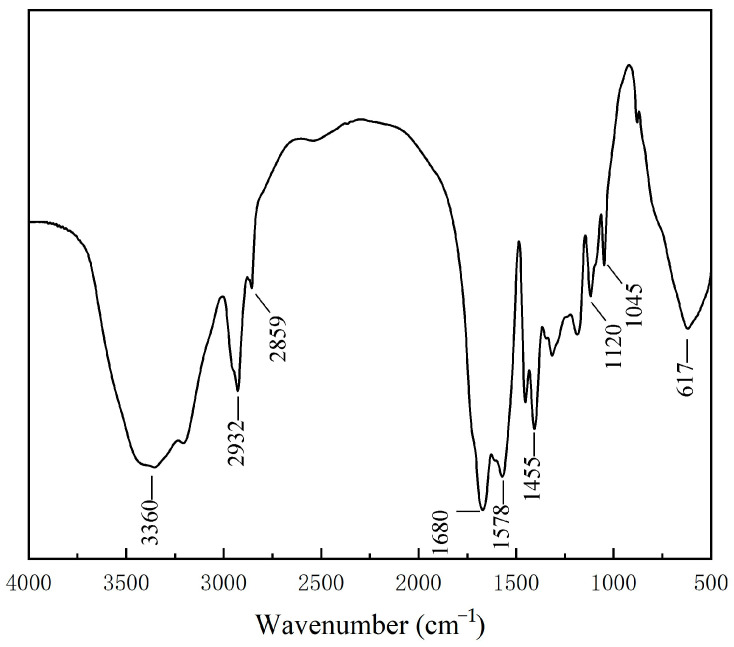
FT-IR spectrum of P(AA-AM-SA)@TiO_2_.

The Fourier Transform Infrared Spectroscopy (FT-IR) spectrum of P(AA-AM-SA)@TiO_2_ microspheres is shown in Figure 6. The peaks at 3360 cm^−1^ and 3210 cm^−1^ are attributed to the N–H stretching vibration of acrylamide and the –OH stretching vibration of TiO_2_ surface, respectively. The peaks at 2932 cm^−1^ and 2859 cm^−1^ are attributed to the symmetric and antisymmetric stretching vibrations of C–H, respectively, which are related to the stretching vibrations of C–H on the six-membered ring of SA macromolecule after Ca^2+^ cross-linking [30]. The peak at 1680 cm^−1^ corresponds to the stretching vibration of the –C=O bond in –COOH [31]. The peak at 1578 cm^−1^ corresponds to the stretching vibration of the –COO^−^ bond. The peak at 1455 cm^−1^ can be assigned to the stretching vibration of the C–N bond and the peak at 1120 cm^−1^ to the C–O stretching vibration in C–OH. The peak at 1045 cm^−1^ corresponds to the C–O stretching vibration in C–O–C, while the peak at 617 cm^−1^ corresponds to the characteristic absorption peak of Ti–O. KH-570 enhances the interfacial compatibility between TiO_2_ and the polymer. By participating in the polymerization reaction through the C=C bond on KH-570, monomers polymerize on the surface of TiO_2_ to form polymer microspheres [32]. The characteristic peaks of AA, AM, and SA observed in the infrared spectrum suggest successful synthesis of the polymer.

#### 2.2.2. Scanning Electron Microscope (SEM) Image Analysis

**Figure 7:**
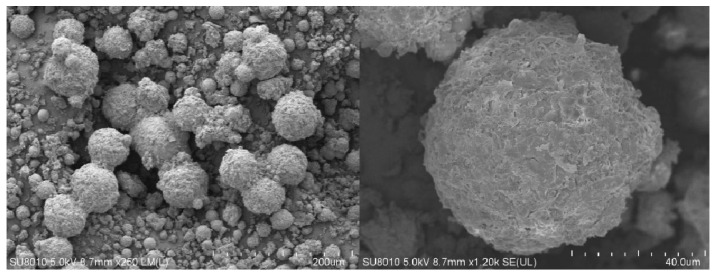
SEM of P(AA-AM-SA)@TiO_2_ at different magnifications.

Figure 7 presents SEM images of P(AA-AM-SA)@TiO_2_ at different magnifications. The microstructure of P(AA-AM-SA)@TiO_2_ appears regular and spherical, with diameters ranging from 10 to 40 μm. As a result of the surface effects, these spheres tend to agglomerate. Notably, the surface of these microspheres demonstrates a rough structure attributed to the coating of alginate.

#### 2.2.3. Energy-Dispersive Spectroscopy (EDS) Analysis

**Figure 8:**
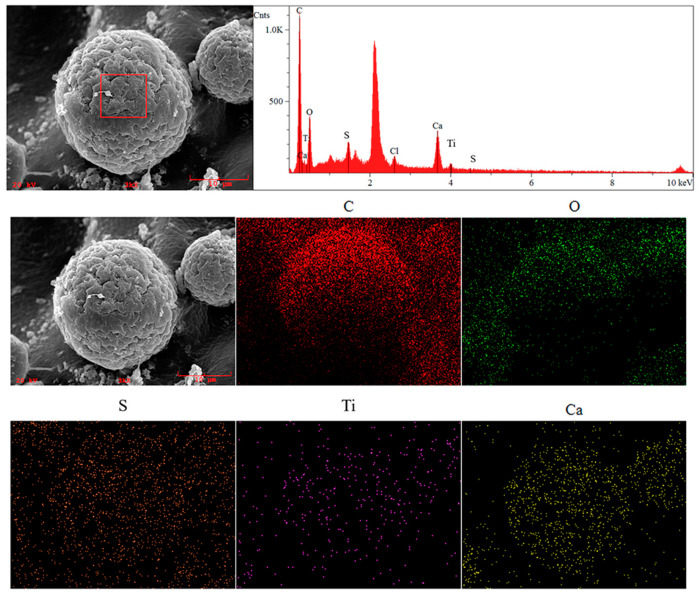
P(AA-AM-SA)@TiO_2_ elemental analysis diagram and elemental distribution diagram.

To further demonstrate the success of synthesizing polymer gel/inorganic nanoparticle microspheres, EDS and EDS-Mapping analyses were conducted on the microsphere samples. As depicted in Figure 8, the P(AA-AM-SA)@TiO_2_ microspheres exhibit a regular morphology, with clear C and O signals indicating the presence of polymer chains within the microspheres. The distribution shape of Ca and Ti element signal points is almost spherical, suggesting that Ca and Ti elements are uniformly distributed in the polymer microspheres, consistent with the expected spherical network structure formed by cross-linking of alginate-calcium. The low quantity of Ti elements in the figure is mainly due to the shielding effect of alginate coating, which hindered its detection.

#### 2.2.4. Thermogravimetric Analysis

**Figure 9:**
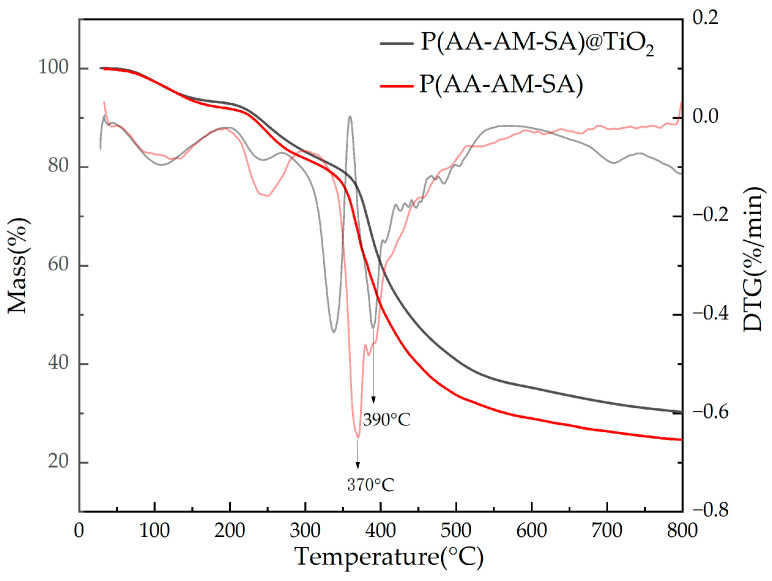
Thermogravimetric curve of P(AA-AM-SA) and P(AA-AM-SA)@TiO_2_.

Figure 9 compares the thermogravimetric analysis of P(AA-AM-SA)@TiO_2_ and P(AA-AM-SA); the weight loss of the P(AA-AM-SA)@TiO_2_ microspheres can be divided roughly into three stages: the first stage involves a mass loss of 6.55% due to the evaporation of water adsorbed on the microspheres; the second stage is characterized by the breakage of the polymer chain, resulting in a mass loss of 13.7%; and, in the third stage, there is a rapid decline in weight loss as the main polymer chain breaks. The weight loss rate during this stage is the highest. The differential thermogravimetric (DTG) results showed that the mass of P(AA-AM-SA) microspheres had a significant loss at 370 °C, while P(AA-AM-SA)@TiO_2_ had a higher decomposition temperature of 390 °C, indicating better thermal stability. This is because, after the addition of KH-570/TiO_2_, TiO_2_ chemically bonds with the polymer, which increases the cross-linking points of the polymer and limits the mobility of polymer chain segments. Thus, the thermal decomposition of the P(AA-AM-SA)@TiO_2_ molecular chain is prevented. Additionally, the uniform distribution of nano-TiO_2_ in the polymer creates a certain barrier effect that inhibits the transfer of heat [33].

### 2.3. Thermal and Aqueous Salinity Resistance

To cope with the complex oil and gas reservoir environment, the thermal and aqueous salinity resistance of microspheres is particularly important.

Firstly, the thermal resistance ability of the microspheres was evaluated. A total of 0.1 g of microsphere powder was added to a sample vial and dispersed in 20 mL of deionized water to prepare a microsphere dispersion. The vial cap was sealed tightly and subjected to ultrasonic dispersion for 30 min. The change in particle size was observed after 3 days of water absorption at different temperatures. Simultaneously, P(AA-AM)@TiO_2_ microspheres prepared without the addition of sodium alginate and P(AA-AM) microspheres prepared without the addition of titanium dioxide were used as controls. The median particle size of the microspheres was tested using a laser particle sizer (Bettersize 2000, Bettersize, Dandong, China).

**Figure 10:**
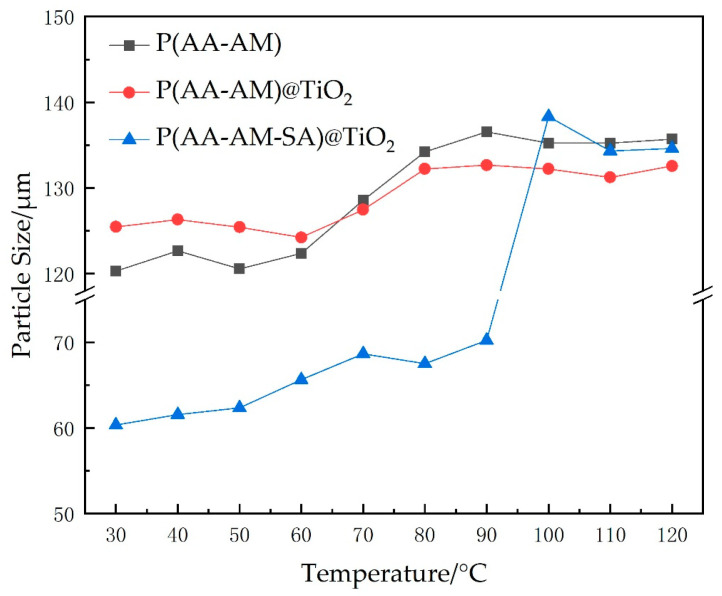
Polymer microsphere expansion particle size variation with temperature.

From Figure 10 below, it can be seen that the size of microsphere P(AA-AM) and P(AA-AM)@TiO_2_ increases continuously with the increase in temperature. The temperature range of 60~90 °C exhibits the maximum variation in particle size, followed by a stabilization trend as the temperature further increases. The increase in temperature enhances the thermal motion of molecules within the polymer chains, consequently facilitating easier penetration of water molecules into the interior of the microspheres. This phenomenon leads to an increase in particle size. Additionally, the rise in temperature increases the osmotic pressure difference across the microsphere, promoting its expansion. Finally, as the temperature further increases, the microspheres do not continue to expand due to their own cross-linking. It is noteworthy that the particle size of P(AA-AM-SA)@TiO_2_ microspheres is comparatively smaller at lower temperatures. However, when the temperature exceeds 90 °C, the particle size rapidly increases, mainly due to the fact that the cross-linking network structure of calcium alginate impedes its water absorption and expansion. Upon reaching a certain temperature, thermal decomposition of calcium alginate releases the internal microsphere structure, causing water absorption and rapid enlargement of the particle size.

Then, NaCl and CaCl_2_ were used to simulate monovalent and divalent salt solution to investigate the salt resistance of the microspheres, and the particle size of water absorption and swelling of the microspheres was tested by laser particle size measurement.

**Figure 11:**
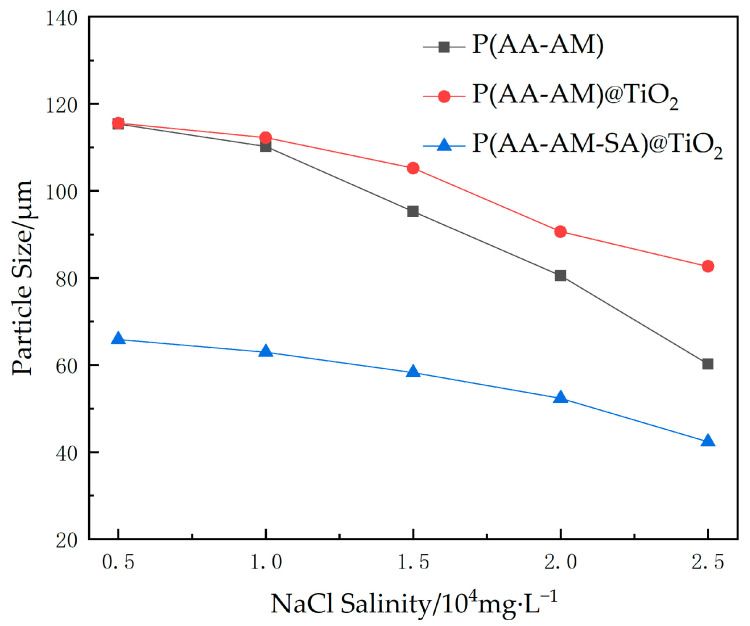
Polymer microsphere expansion particle size variation with different NaCl salinity.

The effects of monovalent salt solutions on polymer microspheres are illustrated in Figure 11. All three types of microspheres exhibited a reduction in their water absorption capacity at higher salt concentrations. This is mainly attributed to the decrease in osmotic pressure inside and outside the microspheres, which hinders water penetration into their interiors. The particle size of P(AA-AM)@TiO_2_ decreases with increasing salt concentration but to a lesser extent than that of P(AA-AM) microspheres. This is mainly due to the addition of TiO_2_, which makes it less susceptible to salt-induced swelling, indicating superior salt resistance compared to P(AA-AM). For P(AA-AM-SA)@TiO_2_, its water absorption capacity is weakened by the presence of salt, while its surface membrane structure weakens its ability to swell in water, resulting in smaller particle size compared to the other two microspheres. However, from the curve in the figure, it can be seen that the effect of salinity on particle size is relatively gentle and, overall, the mineralization has a small effect on particle size within the range of 2.5 × 10^4^ mg/L.

**Figure 12:**
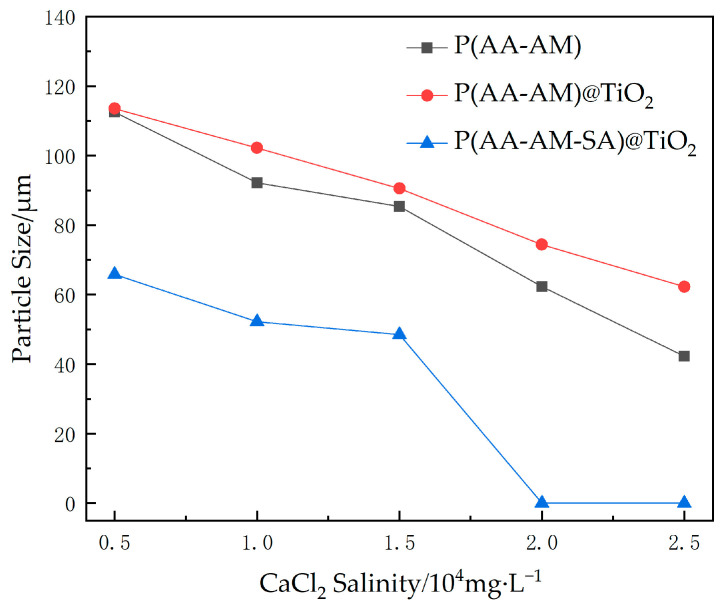
Polymer microsphere expansion particle size variation with different CaCl_2_ salinity.

The results of evaluating the impact of divalent salt solutions on the water absorption of microspheres using CaCl_2_ are shown in Figure 12. Microspheres are significantly affected by divalent ions, primarily due to the higher valence state of Ca^2+^, which has a stronger ability to shield negatively charged microspheres, reducing the electrostatic repulsion between polymer chains and resulting in significant contraction of the chains and weaker water absorption properties.

In particular, P(AA-AM-SA)@TiO_2_ loses almost all of its water absorption ability and aggregates when the CaCl_2_ concentration exceeds 2.0 × 10^4^ mg/L, indicating that the water absorption performance of polymer microspheres coated with calcium alginate is poor in the presence of Ca^2+^. The principle behind this process may involve coating polymer microspheres with calcium alginate following Ca^2+^ cross-linking of sodium alginate, during which some Na^+^ ions may remain unreplaced by Ca^2+^. Therefore, as microspheres swell in high-concentration Ca^2+^ solution, more unexchanged Na^+^ inside the microspheres come into contact with water, leading to further ion gelation and further restriction of polymer microsphere swelling.

In summary, P(AA-AM-SA)@TiO_2_ exhibits good water absorption and swelling performance in NaCl salt solution and maintains good water absorption and expansion performance at concentrations above 2.5 × 10^4^ mg/L. The addition of modified TiO_2_ significantly enhances the mineralization resistance of the microspheres. The water absorption capacity of P(AA-AM-SA)@TiO_2_ is greatly affected by Ca^2+^, and the microspheres are recommended for use in environments where the Ca^2+^ mineralization concentration is below 2.0 × 10^4^ mg/L.

### 2.4. Rheological Properties

In rheology, shear stress and viscosity are commonly used to describe the flow behavior of liquids. Good shear ability and viscoelasticity enhance the transportability of microspheres in porous media. Mechanical strength is an important factor for deep plugging of microspheres, while shear resistance characterizes mechanical strength and reflects the blocking ability inside pores and throats [34,35,36].

**Figure 13:**
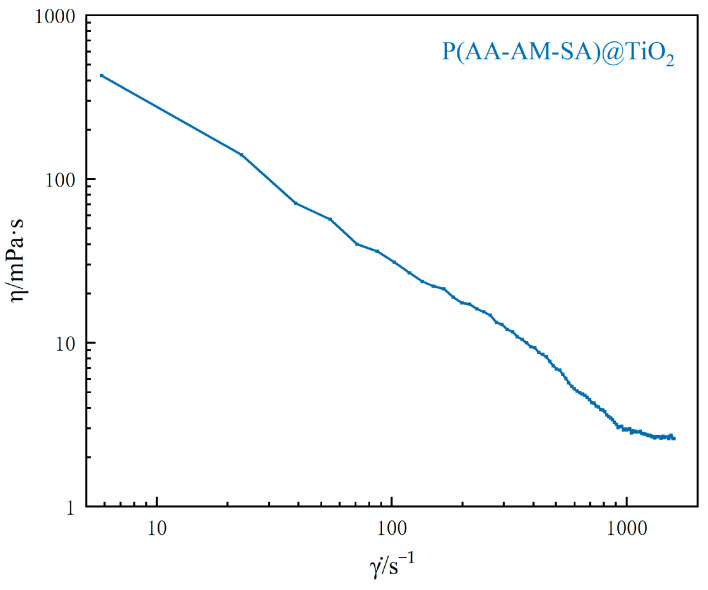
Curve of apparent viscosity with shear rate at 30 °C.

Figure 13 shows the apparent viscosity variation curve of P(AA-AM-SA)@TiO_2_ microsphere dispersion (1000 mg/L) at 30 °C as a function of shear rate. Within the shear viscosity range of 5.8~800 s^−1^, the apparent viscosity of dispersions of microspheres sharply decreases with an increase in shear rate, exhibiting a pseudoplastic behavior. The observed shear-thinning phenomenon is primarily driven by the shear force, which alters the intermolecular forces between the P(AA-AM-SA)@TiO_2_ microspheres, leading to a change in their internal structure and an increase in intermolecular distance, consequently reducing the viscosity of dispersions of microspheres. At shear rates ranging from 800 to 1600 s^−1^, the apparent viscosity of dispersions of microspheres remains virtually constant, exhibiting characteristics similar to those of a Newtonian fluid. This constancy is primarily attributed to the presence of crosslinking agents in the microsphere structure, which, to some extent, limit their deformation and prevent their dissociation into linear chains. When the aggregating and dissociating effects on microspheres induced by shear stress reach a dynamic equilibrium, the shear viscosity of the dispersions of microspheres remains stable, the fluid exhibiting characteristics similar to that of a Newtonian fluid. The above analysis indicates that P(AA-AM-SA)@TiO_2_ microspheres have low viscosity at room temperature, exhibiting a shear-thinning characteristic, which endows it with favorable flowability and excellent injectability, rendering it easy to be injected deep into the formation.

The dynamic modulus is the ratio of stress to strain, which represents the elastic modulus of a material under dynamic loading. It is divided into the storage modulus (G′) and the loss modulus (G″). G′, also known as the elastic modulus, mainly reflects the ability of the material to store energy during elastic deformation, where a larger G′ indicates greater stiffness. G″, also known as the viscous modulus, mainly records the loss of energy due to thermal dissipation during deformation, where a larger G″ generally implies a higher dynamic viscosity of the material and a lower G″/G′ leads to a greater elasticity.

**Figure 14:**
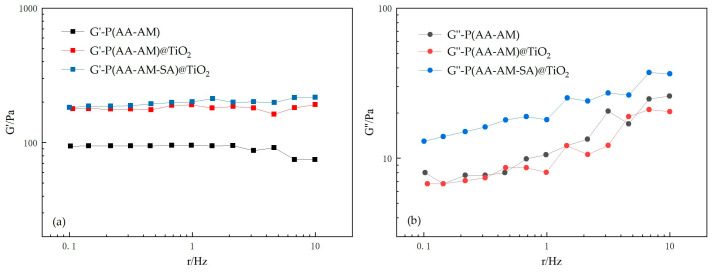
Curves of storage modulus (**a**) and loss modulus (**b**) of dispersions of polymer microspheres with frequency at 30 °C.

Figure 14 shows the frequency-dependent G′ and G″ of three types of dispersions of P(AA-AM-SA)@TiO_2_ microspheres at 30 °C. Overall, the G′ values of three types of dispersions of microspheres are greater than their G″ values, indicating that the microspheres maintain their solid-like behavior at all frequencies [37]. It can be seen that TiO_2_ has a significant impact on the G′ of the microspheres, substantially increasing the elastic modulus of the microspheres. This effect is mainly due to the enhanced elasticity of the microspheres resulting from the interaction between modified TiO_2_ and organic matter, which improves the viscoelastic properties of the microspheres. In general, the higher the G′ and G″ values of microspheres, the better their viscoelastic behavior, indicating a stronger ability to deform and recover their original shape while resisting external shear forces. Incorporating TiO_2_ into polymer microspheres resulted in higher G′ values, enabling them to function as “plugging-migration-plugging” agents during deep migration and achieve both deep migration and plugging capabilities.

**Figure 15:**
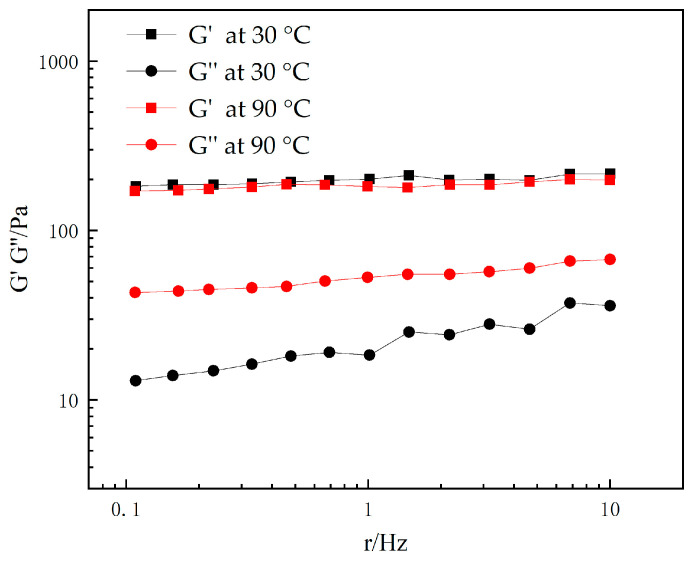
Curves of storage modulus and loss modulus of dispersions of P(AA-AM-SA)@TiO_2_ with frequency at different temperatures.

The viscoelastic properties of P(AA-AM-SA)@TiO_2_ after its water absorption and swelling at high temperature (90 °C) were tested and compared with those at room temperature (30 °C), as shown in Figure 15. The microspheres exhibited a larger G″ at 90 °C, indicating excellent plugging performance in the deep formation at high temperature. On the other hand, the microspheres showed a higher G′ at room temperature, suggesting better elasticity and mobility in the near-wellbore area, without being easily damaged by formation shear. The polymer microspheres showed strong elasticity in the near-wellbore area, facilitating deep migration. As they reached deep in the formation, their strong viscosity contributed to efficient well sealing.

### 2.5. Plugging Performance

The plugging experiment is a crucial method and essential means to evaluate the profile control and water shutoff ability of microspheres. This technique is convenient to carry out and serves as an indoor assessment method commonly employed in the field of enhancing oil recovery (EOR) with the aim of improving the yield rate.

From the profiling control mechanism of microspheres, when the microsphere diameter matches the porosity of the pore, the microspheres can smoothly enter the pore canals and form blockages in the water flooding channel. When the external water flooding pressure is too high, the microspheres deform and pass through the pore, then continue to move to the next throat before blocking again. This process results in fluctuations in pressure. If the microspheres are small, they would pass through the pore throats without blocking and fail to perform their intended function. However, when the microspheres are larger than the pores, it becomes difficult for them to penetrate the rock core, resulting in an increase in pressure and forming an end-face clogging. Therefore, by observing the pressure values, the compatibility between the microspheres and the pore throats can be inferred.

**Figure 16:**
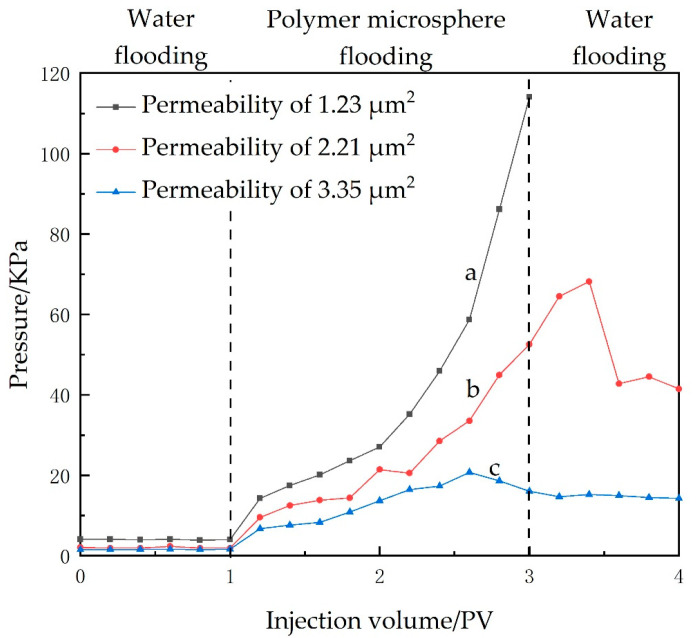
Pressure curves of polymer microspheres injected into different rock cores: (a) Permeability of 1.23 μm^2^; (b) Permeability of 2.21 μm^2^; (c) Permeability of 3.35 μm^2^.

Figure 16 represents the injection profile of microspheres in rock core samples with varying permeabilities, denoted by a, b, and c, having values of 1.23 μm^2^, 2.21 μm^2^, and 3.35 μm^2^, respectively. From the pressure curve shown in Figure 16—a, it can be deduced that there is a gradual increase in pressure without any significant drop at a permeability of 1.23 μm^2^. This indicates the occurrence of end-face clogging, where microspheres failed to inject and cannot effectively plug the area. From the pressure curve of Figure 16—b, it can be inferred that, at the permeability of 2.21 μm², the pressure value increases with the injection of microspheres and decreases during subsequent water flooding. This indicates good compatibility between the microspheres and the rock core. The fluctuation in the pressure curve illustrates a process involving deformation, migration, further deformation, and subsequent migration of the microspheres within the rock core, which suggests that the microspheres can be transported to deep formation. From the pressure curve of Figure 16—c, it can be observed that, when the permeability is 3.35 μm^2^, the pressure slightly increases upon microsphere injection before immediately dropping, indicating that most of the microspheres flowed out and the pressure only exhibits minimal fluctuations. This suggests that the rock core possesses a higher permeability compared to the size of the microspheres used, resulting in poor matching and allowing the microspheres to directly penetrate through the rock core, thus leading to ineffective plugging.

Therefore, for further plugging performance and oil displacement experiments, a core with a permeability of 2.21 μm^2^ was selected. As controls, P(AA-AM)@TiO_2_ microspheres and P(AA-AM) microspheres were used. The experiment was conducted under simulated reservoir conditions using a core length of 15 cm and an internal diameter of 2.5 cm. The experimental process involved water flooding-polymer microspheres flooding-subsequent water flooding, conducted at a temperature of 90 °C.

Table 1 presents the results of plugging experiments. All three types of microspheres exhibited certain plugging abilities. P(AA-AM) microspheres showed relatively inferior plugging performance at 86.4%, primarily due to their weak shear resistance under high-temperature conditions, which hinders their plugging and migration capabilities. The addition of TiO_2_ enhanced the rigidity of the microspheres, and the plugging rate of P(AA-AM)@TiO_2_ microspheres was improved to 94.2%. Regarding the P(AA-AM)@TiO_2_ microspheres, after the degradation of its alginate coating, a portion of calcium alginate is able to enhance the viscoelasticity of the microspheres. Its slow swelling property allows it to be transported to deeper pores, resulting in effective plugging, with a plugging rate of 95.3%.

**Figure 17:**
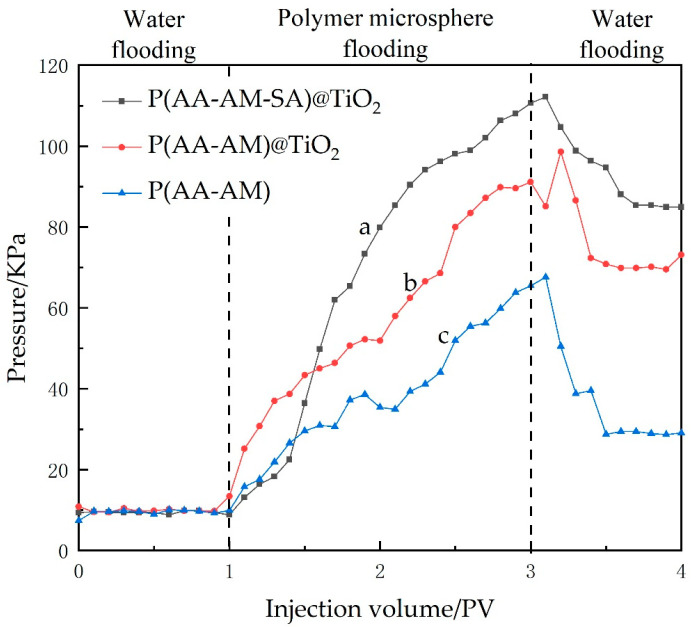
Pressure curves of polymeric microspheres in the oil displacement experiments: (a) P(AA-AM-SA)@TiO_2_; (b) P(AA-AM)@TiO_2_; (c) P(AA-AM).

The injection pressure curves of the three microspheres in the oil displacement experiments are shown in Figure 17. It can be observed from the figure that the injection pressure is low at the beginning of polymer drive, indicating good injectivity of P(AA-AM-SA)@TiO_2_ microspheres; then, the pressure rises to 110 KPa, comparing with the other two microspheres, indicating better plugging performance, and the pressure also maintains a high level in the subsequent water drive phase with better effect. The final computation revealed that the implementation of P(AA-AM)@TiO_2_ microspheres resulted in a significant 10.5% increase in the oil displacement rate. On the other hand, the effect of P(AA-AM) microspheres was comparatively moderate, with only an 8.25% increase in the oil displacement rate. This can primarily be attributed to their diminished resistance to shear at elevated temperatures and weakened plugging performance. In contrast, P(AA-AM-SA)@TiO_2_ microspheres demonstrated the most favorable oil displacement characteristics, achieving 12.89% increase in the oil displacement rate.

## 3. Conclusions

This study involves the synthesis of temperature-sensitive polymer gel/inorganic nanoparticle microspheres, P(AA-AM-SA)@TiO_2_, using a reverse microemulsion polymerization method. Acrylic acid (AA) and acrylamide (AM) were used as co-monomers, while sodium alginate (SA) was incorporated as a temperature-sensitive coating material. Additionally, titanium dioxide (TiO_2_) was included as an inorganic reinforcing agent. Based on the experimental results, the following conclusions can be drawn:

1. Through optimizing the synthetic conditions, uniform polymer gel/inorganic nanoparticle microsphere sizes of 10–40 μm were obtained when the oil-water ratio was 8:5, the emulsifier ratio was 3:1 (10 wt%), the stirring speed was 400 r/min, the reaction temperature was 60 °C, and the initiator dosage was 0.6 wt%.
2. Through the utilization of various analytical tools, such as SEM, EDS, EDS-mapping, FT-IR, and TGA, the structure and morphology of P(AA-AM-SA)@TiO_2_ microspheres was performed. It was revealed by FT-IR that these microspheres possess Ti-O chemical bonds, and EDS along with EDS-mapping confirmed the presence of Ti and Ca elements, indicating synthesis success. Additionally, the SEM results demonstrate a distinct P(AA-AM-SA)@TiO_2_ microsphere coating structure, while TGA shows that P(AA-AM-SA)@TiO_2_ has good thermal stability, and the larger thermal weight loss occurs at 390 °C, which can adapt to the deep formation environment.
3. Through testing for properties such as high-temperature and salt resistance, rheology, and viscoelasticity, the P(AA-AM-SA)@TiO_2_ microspheres exhibited good high-temperature and salt resistance, with sodium salt resistance up to 2.5 × 10^4^ mg/L and calcium salt resistance up to 2.0 × 10^4^ mg/L. The coating rupture temperature of P(AA-AM-SA)@TiO_2_ microspheres at high aqueous salinity was 90 °C. The dispersion of P(AA-AM-SA)@TiO_2_ microspheres exhibits shear-thinning behavior, making it easy to inject into the formation. Under high shear conditions, it maintains nearly Newtonian fluid properties, enabling it to achieve plugging capabilities.
4. Through the flooding experiments, the injectivity, plugging performance, and oil displacement effectiveness of polymer gel/inorganic nanoparticle microspheres were evaluated. Polymer gel/inorganic nanoparticle microspheres exhibited good injectivity in permeabilities ranging from 1.23 to 2.21 μm^2^. At a permeability of 2.21 μm^2^, a plugging efficiency of 95.3% was achieved with an oil displacement rate of 12.89%. This is mainly because the microspheres are first transported to the deep part of the rock core and then expand and plug the pores after coating rupture in a high-temperature environment. Therefore, they have broad application prospects.

## 4. Materials and Methods

### 4.1. Materials

Acrylamide (AM), acrylic acid (AA), N,N-methylenebisacrylamide (MBA), ammonium persulfate (APS), sodium bisulfite (SHS), Span-80, and Tween-80 were purchased from Shanghai Aladdin Biochemical Technology Co., Ltd. (Shanghai, China); sodium hydroxide (NaOH), sodium alginate (SA), titanium dioxide (TiO_2_), and 3-methacryloxypropyltrimethoxysilane (KH-570) were purchased from Shanghai Macklin Biochemical Co., Ltd. (Shanghai, China). Deionized water was made from laboratory water purification equipment.

### 4.2. Experiment Methods

#### 4.2.1. Synthesis of KH570/TiO_2_

**Figure 18:**
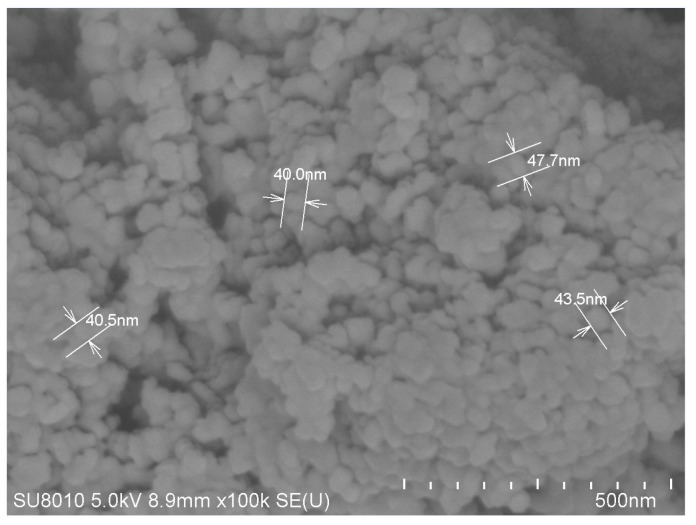
SEM image of nano-TiO_2_.

Firstly, the nano-TiO_2_ was placed in a thermostatic drying chamber and dried at 90 °C for 12 h. A total of 2.0 g of dried nano-TiO_2_ was added into a 250 mL three-neck flask and dispersed with ultrasonication for one hour. Then, 0.8 g of KH-570 and a 3:1 (*w*/*w*) mixture of ethanol and water were added to the flask. The pH was adjusted to 4, the mixture heated to 80 °C, and an ultrasonic reaction conducted for 3 h. The precipitate was centrifuged for 5 min using a centrifuge, rinsed with anhydrous ethanol, and then centrifuged again for 5 min. After repeating centrifugation-rinsing three times, the precipitate was dried at 80 °C for 12 h and ground to powder form to obtain KH-570-modified TiO_2_. The SEM image of nano-TiO_2_ is shown in Figure 18.

#### 4.2.2. Synthesis of Polymer Microspheres

The synthesis of polymer gel/inorganic nanoparticle microspheres was as follows: firstly, solvent cyclohexane was added along with a 3:1 mass ratio of Span80 and Tween80 into a three-necked flask of 250 mL. The oil phase was uniformly dispersed through mechanical stirring in a water bath at 30 °C. Next, AA (2.8 g), AM (2.2 g), MBA (0.75 g), and KH-570/TiO_2_ (0.67 g) were dissolved into deionized water and the pH adjusted to 7 with NaOH. Then, SA (0.5 g) was dissolved in deionized water, and then the aqueous phase was slowly added dropwise into a three-necked flask containing the oil phase under a nitrogen atmosphere. The mixture was stirred at 400 r/min for 30 min using a mechanical stirrer. After that, APS (0.15 g) and SHS (0.15 g) were added to the three-neck flask to initiate the reaction and the temperature was increased to 60 °C for two hours to obtain the polymeric microsphere dispersion emulsion. Anhydrous ethanol was utilized to break up the emulsion of dispersion emulsion, and the P(AA-AM-SA)@TiO_2_ microsphere powder was obtained by extraction, washing with anhydrous ethanol, drying, and grinding.

#### 4.2.3. Characterization Methods of Microspheres

The particle size of microspheres was tested using Laser Particle Size Analyzer (Bettersize 2000, Bettersize, Dandong, China). The powder sample was added to the sample cells, the shading ratio was kept greater than 6%, and the sample was dispersed into water by ultrasound to test the median particle size.

Infrared characterization was performed using the FTIR spectrometer (Vertex 80v, Bruker, Berlin, Germany) and samples were prepared by the KBr tablet method (1 mg polymer microspheres mixed with 100 mg KBr).

The morphology of the polymer microspheres was observed using the SEM (SU8010, Hitachi, Tokyo, Japan). A small amount of samples was adsorbed on the conductive gel, then the part that came off easily was blown, sprayed with gold, and then tested. Moreover, SEM was used for EDS and EDS-Mapping analysis.

The thermal stability of polymer microspheres was studied using the thermogravimetric analyzer (TG/DSC1, Hitachi, Tokyo, Japan). The thermal stability was tested in an argon atmosphere by ramping up from 30 °C to 800 °C at a rate of 15 °C/min.

#### 4.2.4. Rheological Evaluation

The rheology of the dispersions of microspheres, along with the viscoelasticity, was tested using the rheometer (Mars60, HAAKE, Vreden, Germany). Microspheres were added to mineralized water, with a mineralization degree of 1.5 × 10^4^ mg/L (1.0 × 10^4^ mg/L for NaCl; 0.5 × 10^4^ mg/L for CaCl_2_) to prepare the microsphere dispersion with a microsphere mass concentration of 1000 mg/L. The three microspheres were first swollen at 30 °C and high mineralization for 3 d. The test time was set to 200 s to observe the rheological properties of the microsphere solutions at shear rates ranging from 5.8 to 1600 s^−1^.

Then, the relationship between G′ and G″ with frequency for the three microspheres was also studied at 30 °C from 0.1 Hz to 10 Hz; the amplitude of the strain was 10%. Additionally, viscoelasticity was tested for P(AA-AM-SA)@TiO_2_ microspheres at 90 °C and compared to results obtained at 30 °C. To prevent moisture evaporation from the sample during testing, the fixture was adjusted to the desired gap and excess sample removed from the edges. Then, silicone oil was used to seal the gap between the fixture and the sample, achieving a liquid seal effect.

#### 4.2.5. Plugging Performance Test

**Figure 19:**
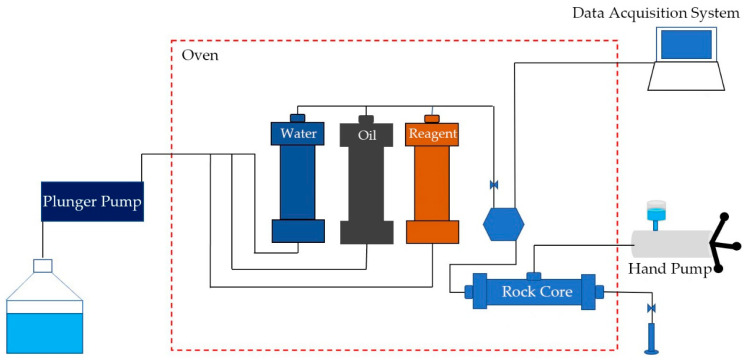
Diagram of plugging performance test equipment.

The schematic diagram of the plugging performance evaluation device for polymer gel microspheres is shown in Figure 19, which can be used to test the injectivity, permeability, and plugging performance of the microspheres, as well as the oil displacement performance. The sand-filled pipe used in the experiment was 15 cm long, with an inner diameter of 2.5 cm. The plugging performance and oil displacement experiments were divided into water flooding-polymer flooding-subsequent water flooding, and the temperature of the experiment was 90 °C. The total concentration of 1.5 × 10^4^ mg/L (1.0 × 10^4^ mg/L for NaCl; 0.5 × 10^4^ mg/L for CaCl_2_) of mineralized water was prepared for the water drive as well as the preparation of microsphere dispersions.

The plugging experiment followed these steps: the rock core dry weight was weighed after drying it at 120 °C for 6 h; then, the core was sealed in a vacuum filtering bottle and saturated with mineralized water for 6 h to obtain the wet weight. The porosity of the rock core was calculated based on the dry weight and wet weight. Next, the equipment was assembled and mineralized water, crude oil, and microsphere dispersions introduced successively into the water tank, oil tank, and reagent tank. A confining pressure of 4 MPa was applied to the rock core; then, the flow rate of the plunger pump was set at 1.0 mL/min to inject mineralized water into the rock core. The stable injection pressure was recorded to determine the initial permeability, K_1_. Then, the microsphere dispersion was injected at a displacement rate of 1.0 mL/min and an injection volume of 2.0 PV (pore volume). Finally, water flooding was performed at 1.0 mL/min until stable pressure was reached to calculate K_2_, the blocked permeability after microsphere plugging, and then the plugging ratio was calculated.

The oil displacement experiment followed these steps: after obtaining initial permeability values using the same approach, crude oil was injected into the core through the displacement pump. The volume of produced water was measured once the crude oil appeared and was left to age for 12 h under simulated reservoir conditions. Mineralized water was then injected into the core through a displacement pump until a water saturation of 98% or more was achieved and the water no longer displaced oil. Polymer microspheres were subsequently injected into the core at a volume of 2.0 PV. A subsequent water flooding was performed until no more oil was produced, collected pressure change values during the displacement process, recording oil and water production amounts, and calculating the recovery ratio. The flow rate of the entire process was controlled at 1.0 mL/min.

## Figures and Tables

**Table 1:** Results of plugging experiments.

Microsphere Types	Permeability K_1_/μm^2^	Permeability K_2_/μm^2^	Plugging Rate/%
a: P(AA-AM-SA)/TiO_2_	2.21	0.102	95.3
b: P(AA-AM)/TiO_2_	2.20	0.127	94.2
c: P(AA-AM)	2.21	0.300	86.4

## Data Availability

Not applicable.
